# Enhanced Dissolution and Oral Bioavailability of Tegoprazan Using D-α-Tocopheryl Polyethylene Glycol 1000 Succinate-Based Melt Granulation System

**DOI:** 10.3390/molecules31193382

**Published:** 2026-09-23

**Authors:** Min Gyeong Jeong, Tae Hyoung Kim, In Gyu Yang, Yeon Ju Baek, Min-Koo Choi, Myung Joo Kang, Ji Hoon Jeong

**Affiliations:** 1 College of Pharmacy, Dankook University, 119 Dandae-ro, Dongnam-gu, Cheonan, Chungnam 31116, Republic of Korea; lmo4555@naver.com (M.G.J.); yeonju7225@dankook.ac.kr (Y.J.B.); minkoochoi@dankook.ac.kr (M.-K.C.); 2 Department of MetaBioHealth, School of Medicine, Sungkyunkwan University, Suwon 16419, Republic of Korea; gene3kim@gmail.com (T.H.K.); dhakflzk@naver.com (I.G.Y.); 3 School of Pharmacy, Sungkyunkwan University, Suwon 16419, Republic of Korea

**Keywords:** tegoprazan, melt granulation, TPGS, stability, dissolution, oral bioavailability

## Abstract

Tegoprazan (TPZ) is a promising potassium-competitive acid blocker, but its pH-dependent low solubility often leads to erratic oral absorption. Although several hydrophilic polymer-based amorphous solid dispersions have been attempted to enhance dissolution, they often suffer from thermodynamic instability or rely on complex, solvent-mediated manufacturing processes. Herein, to enhance dissolution and oral absorption, a crystalline drug-loaded melt granulation (MG) system using D-α-tocopheryl polyethylene glycol 1000 succinate (TPGS) was fabricated while maintaining stable physicochemical properties. MGs were prepared via a solvent-free, hot-melt process by optimizing the type of solid surfactant carrier, the ratio of drug to solid surfactant, and the type and ratio of flow-enhancing excipients. The optimized composition was TPZ:TPGS:microcrystalline cellulose = 1:2:4 (*w*/*w*/*w*). Solid-state analyses revealed that TPZ was uniformly dispersed within the granules while retaining its crystalline form. Compared to the raw drug, the MG showed remarkably faster and higher dissolution across both acidic and neutral media. Under long-term (25 °C/60% RH) and accelerated storage conditions (40 °C/75% RH) for 24 weeks, MG exhibited robust physicochemical stability, showing no drug degradation, changes in dissolution behavior, or crystalline alterations. Moreover, in a pharmacokinetic evaluation in Sprague–Dawley rats, MG provided marked absorption enhancement, yielding over 1.8-fold increases in exposure compared to the raw material. Consequently, the crystalline drug-loaded TPGS-based MG represents a promising strategy for improving the oral delivery of TPZ.

## 1. Introduction

Tegoprazan (TPZ; 7-(((2*R*,4*S*)-5,7-difluorochroman-4-yl)oxy)-*N*,5-dimethyl-1*H*-benzo[d]imidazole-2-carboxamide) is a novel, potent potassium-competitive acid blocker that provides rapid, strong, and reversible inhibition of the gastric H^+^/K^+^-ATPase enzyme complex [1,2]. Compared to traditional proton pump inhibitors, TPZ offers distinct clinical advantages, including a rapid onset of action, meal-independent efficacy, and prolonged suppression of nocturnal acid breakthrough [3,4,5,6]. TPZ is a weakly basic drug with a pK_a_ of 5.1 and a lipophilic octanol–water partition coefficient (log P ~ 2.4) [7,8]. It exhibits high membrane permeability but pH-dependent and inherently poor aqueous solubility (<0.05 mg/mL at neutral pH) [7]. Consequently, the incomplete dissolution of TPZ in the gastrointestinal tract restricts its intestinal absorption, leading to low and erratic oral absorption [9]. Although approved oral formulations—such as conventional film-coated or disintegrating/dispersible tablets—enhance patient compliance, they primarily facilitate rapid disintegration rather than intrinsic solubility enhancement [3,10]. Consequently, upon reaching the higher pH environment of the small intestine, TPZ released from these formulations suffers from severe precipitation, leading to erratic absorption kinetics and high pharmacokinetic variability [7,8].

To overcome the dissolution hurdles of poorly water-soluble drugs, solid dispersion technology has been widely investigated, including for TPZ [9,11]. Previous studies have primarily focused on amorphous solid dispersions (ASDs), using hydrophilic polymers to disperse TPZ at the molecular level through solvent-based techniques like spray drying or fluid-bed coating [9,12]. Actually, the ASD prepared with hydroxypropyl methylcellulose (HPMC) or polyvinylpyrrolidone K17 (PVP K17) showed significant absorption enhancement, achieving up to a 2.1-fold increase in oral absorption compared to the raw material [9]. While ASDs significantly improve drug dissolution by bypassing the crystal lattice energy barrier and facilitating microenvironmental wettability [12], their thermodynamically unstable nature predisposes them to phase separation and recrystallization, ultimately impairing physical stability over extended storage [10,12]. Moreover, solvent-based manufacturing is hindered by process complexity, high operational costs, and safety hazards regarding residual organic solvent toxicity and environmental sustainability [12].

To overcome these manufacturing and stability constraints, a melt granulation (MG) system utilizing solid surfactants with melting points above 30 °C has emerged as an attractive, solvent-free alternative for solid dispersion preparation [13,14]. In this process, amphiphilic carriers that remain solid at room temperature but melt at mild processing temperatures—such as D-α-tocopheryl polyethylene glycol 1000 succinate (TPGS; melting point (T_m_) ~ 37–41 °C), Poloxamers (e.g., Poloxamer 188, T_m_ ~ 52 °C; Poloxamer 407, T_m_ ~ 53–57 °C), and Gelucire grades (e.g., Gelucire 44/14, T_m_ ~ 42.5–47.5 °C; Gelucire 50/13, T_m_ ~ 46–51 °C)—are thermally processed without organic solvents [13,15,16]. Previous studies have demonstrated that MG systems successfully enhance the oral absorption of various poorly water-soluble drugs; for instance, TPGS- or Gelucire-based MGs of fenofibrate, celecoxib, and carvedilol led to marked improvements in dissolution rates and multi-fold increases in oral bioavailability [14,15,17]. By adjusting the composition and fabrication process, solid surfactant-based MG allows the drug to remain uniformly dispersed within a hydrophilic or amphiphilic matrix while preserving its thermodynamically stable crystalline state [17,18]. This crystalline dispersion strategy provides superior long-term physical and chemical stability against recrystallization, while simultaneously conferring dramatic solubility/dissolution enhancement through surfactant-mediated wicking, reduction in the diffusion boundary layer, micellar solubilization, and improved microenvironmental wettability [16,19].

In the present study, we engineered a novel MG system containing TPZ in its crystalline form to achieve improved dissolution and oral absorption, while providing excellent physicochemical stability. Various solid amphiphilic carriers (TPGS, Poloxamer, and Gelucire) alongside flow-enhancing excipients were screened to enhance drug dissolution. The solid-state properties and morphology of TPZ within the MG system were characterized using scanning electron microscopy (SEM), powder X-ray diffraction (PXRD), and differential scanning calorimetry (DSC). Furthermore, in vitro dissolution behavior was comparatively evaluated across physiological pH environments. Finally, the in vivo pharmacokinetic (PK) profile of TPZ following oral administration of the MG was evaluated in rats in comparison with raw drug powder.

## 2. Results and Discussion

### 2.1. Formulation Development and Selection of Solid Surfactant Carriers

Solid dispersion techniques are widely utilized to enhance the dissolution rate and oral bioavailability of poorly water-soluble drugs. Typically, ASDs offer a distinct advantage in apparent solubility by eliminating the crystal lattice energy barrier [12]. However, the high thermodynamic energy state of amorphous drugs renders them highly susceptible to phase separation and recrystallization during storage under stress conditions, thereby compromising their long-term physical stability [11,12]. In contrast, crystalline solid dispersion approaches using hydrophilic or amphiphilic polymeric carriers maintain the drug in a stable crystalline or partially crystalline state within a protective carrier matrix [18]. Specifically, MG is widely employed to prepare such crystalline solid dispersions; it is a solvent-free process in which the active drug is uniformly dispersed in a molten carrier and subsequently cooled in combination with pharmaceutical excipients [13,18,19]. This strategy circumvents thermodynamic instability while substantially improving drug dissolution through carrier-mediated wetting enhancement, local micellar solubilization, and the prevention of drug particle agglomeration [16,18,19,20]. Therefore, a solvent-free hot-melt granulation method was selected in this study to construct a physically stable and dissolution-enhanced formulation for TPZ.

To select the effective surfactant carrier for the MG, the equilibrium solubility of TPZ was evaluated in a phosphate buffer (pH 6.8) containing 1% (*w*/*v*) of solid surfactants (Figure 1).

In the absence of surfactants, TPZ exhibited low aqueous solubility (45.1 ± 0.8 µg/mL) at pH 6.8 due to its unionized fraction [7]. However, the incorporation of amphiphilic surfactants above their critical micelle concentrations markedly increased the apparent solubility of TPZ through hydrophobic encapsulation within the core of self-assembled micellar structures [21,22]. Among the tested surfactants, TPGS displayed the highest solubilizing capacity for TPZ (318.4 ± 31.2 µg/mL), achieving an approximately 7.1-fold increase in solubility compared to the raw material. Gelucire 44/14 (269.1 ± 3.2 µg/mL), Gelucire 50/13 (264.8 ± 7.1 µg/mL), and Kolliphor RH 40 (268.0 ± 0.9 µg/mL) also showed substantial solubility enhancements (approximately 5.9- to 6.0-fold increases), whereas Poloxamer 188 (74.9 ± 0.8 µg/mL) and Poloxamer 407 (74.8 ± 8.9 µg/mL) exhibited relatively modest effects. TPGS is a non-ionic, amphiphilic, and biocompatible surfactant formed by esterifying vitamin E succinate with polyethylene glycol 1000 [23]. Beyond its superior micellar solubilization capacity [21,22], TPGS possesses advantageous physicochemical properties as an MG carrier, including a low T_m_ of approximately 37–41 °C [24], high safety profile [25], and known P-glycoprotein efflux inhibition capability, which can potentially enhance oral bioavailability [26,27]. Considering both its superior solubilization capacity and multifunctional physicochemical advantages, TPGS was selected as the primary amphiphilic matrix for preparing TPZ-loaded MGs.

### 2.2. Formulation and In Vitro Dissolution of TPZ-Loaded MGs

To establish the most suitable composition of the TPZ-loaded MG system, the dissolution profiles of MGs as functions of drug-to-TPGS ratios (F1–F4), carrier types (F2 and F5–F8), and microcrystalline cellulose (MCC) amounts (F2, F9, and F10) were evaluated in phosphate buffer at pH 6.8. This dissolution medium was selected because it mimics the physiological environment of the small intestine—the primary site of drug absorption—where weakly basic drugs such as TPZ exhibit inherently low aqueous solubility [7]. The hot-melt granulation process was conducted at an elevated temperature of 70 °C. This specific temperature was chosen to ensure the complete liquefaction of TPGS (T_m_ ~ 37–41 °C) and a marked reduction in molten matrix viscosity, thereby facilitating homogeneous dispersion of the active drug and excipients while preserving the thermal stability of TPZ. Under these processing conditions, TPZ remained chemically stable, with no detectable drug loss or degradation product formation. The compositions of all prepared TPGS-based formulations are summarized in Table 1.

F1–F4 were designed to evaluate the effect of the drug-to-surfactant (TPZ:TPGS) ratio. F2 and F5–F8 were prepared to compare the impact of different pharmaceutical excipients (MCC, HPMC, PVP K17, sodium carboxymethyl cellulose (Na-CMC), and Aerosil 200) for improving the flowability and medium dispersibility of MGs, while keeping the drug-to-surfactant ratio constant (1:2). Subsequently, F2, F9, and F10 were evaluated to determine the appropriate proportion of MCC.

#### 2.2.1. Effect of TPGS Content (F1–F4) on Dissolution Profile of TPZ MGs

The effect of the amount of TPGS (50, 100, 150, and 200 mg) on the dissolution profile of TPZ (50 mg) was evaluated using formulations F1 through F4, which contained drug-to-TPGS weight ratios ranging from 1:1 to 1:4 (Figure 2a).

Increasing the proportion of TPGS significantly enhanced both the dissolution rate and the extent of drug release compared to drug powder. F1 (1:1) reached a maximum drug release of only approximately 66.0% at 120 min. Increasing the TPGS ratio to 1:2 (F2) resulted in a pronounced enhancement in dissolution, achieving 71.4% release within 10 min and reaching 84.0% at 120 min. Further increases in TPGS content (F3 [1:3] and F4 [1:4]) provided incremental improvements, achieving 91.9% and 93.0% release at 120 min, respectively.

This dose-dependent enhancement in dissolution can be attributed to the amphiphilic nature of TPGS, which markedly lowers the interfacial tension between the hydrophobic TPZ surface and the aqueous dissolution medium, thereby facilitating rapid wetting and water penetration [28,29]. Furthermore, at TPGS concentrations exceeding its critical micelle concentration, self-assembled micellar structures effectively encapsulate TPZ molecules within their hydrophobic core, preventing rapid recrystallization or precipitation during release [28,30]. While F2 displayed a substantial jump in dissolution performance relative to F1, the additional dissolution gains observed in F3 and F4 were comparatively modest relative to the increased mass of surfactants. Excessive surfactant levels are generally undesirable in solid dosage forms, as high concentrations of surfactants can induce potential mucosal irritation in the gastrointestinal tract and unnecessarily increase the overall bulk volume of the final formulation [31]. Considering both the dissolution efficiency and formulation toxicity/bulkiness, the 1:2 weight ratio of TPZ:TPGS (F2) was considered as the ideal drug-to-surfactant foundation for formulating TPZ MGs.

#### 2.2.2. Influence of Flow-Enhancing Excipients on the Dissolution Profile of TPZ MGs

To enhance the flowability, physical integrity, and spreading in the dissolution medium of the MG system, different hydrophilic polymers and inorganic adsorbents (MCC, HPMC E50, PVP K17, Na-CMC, and Aerosil 200; 100 mg each) were incorporated while maintaining the TPZ:TPGS ratio at 1:2 (F2 and F5–F8, Figure 2b). The inclusion of these functional excipients markedly improved the powder flowability and mechanical resistance of the MGs. In particular, hydrophilic carriers accelerated media penetration and facilitated rapid spreading upon contact with aqueous dissolution media. This enhanced wettability effectively prevented localized agglomeration, thereby enabling rapid disintegration and uniform drug release.

A distinct disparity in dissolution behavior was observed depending on the physicochemical properties of the added carriers. Formulations containing MCC (F2), PVP K17 (F6), and Aerosil 200 (F8) displayed rapid and complete dissolution profiles, achieving 79.6%, 81.5%, and 81.1% release at 120 min, respectively. Among these, the initial release was particularly rapid for F2, reaching 63.1% at 10 min. MCC (F2) functions as an insoluble yet highly porous carrier matrix featuring strong intraparticle capillary action (wicking effect). Upon exposure to the dissolution medium, MCC rapidly draws water into the interior of the melt granules through capillary forces, promoting immediate matrix breakdown and facilitating the rapid dispersion of TPGS-solubilized TPZ droplets into the aqueous medium without forming viscous hydrogel barriers [32,33]. In contrast, formulations containing swellable hydrophilic polymers, namely HPMC (F5) and Na-CMC (F7), displayed markedly retarded dissolution, achieving only 50.7% and 60.7% cumulative drug release at 120 min, respectively. The marked reduction in dissolution rate observed with HPMC (F5) and Na-CMC (F7) is attributed to the formation of a viscous hydrogel layer upon hydration around the granule surface. This gel membrane creates a robust diffusional barrier that hinders water entry into the granule core and retards the diffusion of micelle-solubilized TPZ into the bulk medium [34]. Taken together, MCC was selected as the primary functional carrier material for further formulation refinement.

#### 2.2.3. Effect of MCC Amount on Dissolution Profile of TPZ MGs

To evaluate the appropriate proportion of MCC, the TPZ:TPGS ratio was fixed at 1:2 (50 mg TPZ, 100 mg TPGS), and the MCC content was varied from 100 mg to 300 mg (F2, F9, and F10, corresponding to drug:TPGS:MCC weight ratios of 1:2:2, 1:2:4, and 1:2:6; Table 1, Figure 2c). As the MCC content increased from 100 mg (F2) to 200 mg (F9), initial dissolution at 10 min significantly improved from 62.2% (F2) to 72.0% (F9), with total cumulative release at 120 min increasing from 77.4% to 85.5%. Further increasing the MCC content to 300 mg (F10) yielded a slight additional improvement, achieving 78.6% release at 10 min and 89.3% at 120 min.

The enhanced drug release with increasing MCC levels is attributed to the larger total surface area available for drug dispersion and a reinforced wicking action, which accelerates granule breakdown and prevents particle agglomeration during dissolution [33,35]. However, the marginal dissolution gain of F10 over F9 was relatively small (~4 percentage-point increase at 120 min) and comes at the expense of significantly increasing the total unit dosage mass (final unit weight of 450 mg for F10 vs. 350 mg for F9). Taking into consideration both the dissolution efficiency and the practical requirement of minimizing final oral dosage size to facilitate patient compliance, formulation F9 (TPZ:TPGS:MCC = 1:2:4 (*w*/*w*/*w*)) was finalized as the target TPZ MG system for subsequent characterization and in vivo PK evaluations.

### 2.3. In Vitro Dissolution Profile of TPZ MG Across Various Media

To evaluate the gastrointestinal dissolution behavior of TPZ from the finalized MG, in vitro dissolution studies were conducted using the USP Apparatus II (paddle method, 50 rpm) across four distinct dissolution media: simulated gastric fluid (pH 1.2), acetate buffer (pH 4.0), phosphate buffer (pH 6.8), and distilled water (DW) in comparison with both intact TPZ raw material and the commercial film-coated tablet (Figure 3a–d). Because TPZ is a weakly basic drug, its equilibrium solubility exhibits strong pH dependence, decreasing drastically as the media pH transitions from acidic to neutral conditions (solubility: >10 mg/mL at pH 1.2, ~0.5 mg/mL at pH 4.0, and <0.05 mg/mL at pH 6.8 and DW) [7,10]. Thus, evaluating the dissolution profiles across these diverse pH environments is essential to understand the formulation performance in the gastrointestinal tract.

In simulated gastric fluid (pH 1.2), TPZ raw material, commercial film-coated tablet, and TPZ MG all exhibited rapid and complete drug release within the initial 10 min, achieving cumulative dissolution rates exceeding 90.0% (Figure 3a). The weakly basic TPZ molecules undergo extensive protonation, yielding high aqueous solubility in the simulated gastric fluid. A slight decline in drug concentration observed toward 120 min is likely attributable to the inherent chemical instability of TPZ in strongly acidic environments [36]. On the other hand, in acetate buffer (pH 4.0), both TPZ MG and the commercial tablet displayed rapid dissolution, reaching over 80.0% dissolution within 10 min and progressing to 92.1% and 91.5% at 120 min, respectively (Figure 3b). In contrast, intact TPZ raw material exhibited markedly slower dissolution kinetics, achieving only 26.5% release at 5 min and requiring 120 min to reach 84.1%. The reduced degree of drug ionization at pH 4.0 compared to pH 1.2 resulted in a lower dissolution rate and extent for raw TPZ due to the absence of strong protonation. Conversely, the rapid initial release of both formulated groups (MG and commercial tablet) highlights their formulation advantages over unformulated API: while the commercial tablet utilizes a conventional wet-granulated matrix incorporating hydrophilic fillers and disintegrants (e.g., microcrystalline cellulose, D-mannitol, hydroxypropyl cellulose, colloidal silicon dioxide, and croscarmellose sodium) to facilitate rapid disintegration and effective particle wetting, TPZ MG achieves rapid dissolution through the immediate wetting action of TPGS [37] and the capillary wicking of the porous MCC carrier [33].

Under intestinal conditions (pH 6.8 phosphate buffer) and unbuffered aqueous media, where TPZ exhibits severely restricted baseline solubility, the formulation advantage of TPZ MG was most pronounced (Figure 3c,d). In pH 6.8 phosphate buffer (Figure 3c), intact TPZ raw material suffered from severe dissolution hindrance due to its unionized hydrophobic state [10], exhibiting a poor initial dissolution of only 2.8% at 5 min and reaching a cumulative release of merely 29.4% at 120 min. The commercial tablet exhibited superior dissolution over the raw API (35.1% at 5 min; 59.8% at 120 min) in pH 6.8 buffer. Superdisintegrants (croscarmellose sodium) and hydrophilic additives facilitate rapid tablet breakdown into micronized primary particles, thereby increasing the effective surface area and microenvironmental wettability. TPZ MG provided superior dissolution efficiency in pH 6.8 buffer, achieving 46.8% release at 5 min and progressing to 68.9% at 120 min. This represents a substantial improvement in both early dissolution velocity and cumulative drug release over the commercial tablet (35.1% at 5 min; 59.8% at 120 min) as well as the raw API (2.8% at 5 min; 29.4% at 120 min) across all time points. A similar trend was also observed in DW (Figure 3d), where intact TPZ raw material showed limited dissolution performance, releasing only 7.0% at 5 min and plateauing at 34.0% at 120 min, whereas TPZ MG displayed superior dissolution kinetics, achieving 47.0% within 5 min and progressing to 68.9% at 120 min (>6.7-fold at 5 min and >2.0-fold at 120 min). In DW, the commercial tablet exhibited a dissolution profile comparable to that of the TPZ MG system.

This dramatic dissolution enhancement in solubility-restricted environments (pH 6.8 and DW) relies on a synergistic interplay of three key formulation factors: surfactant-mediated wetting, micellar solubilization, and capillary wicking-induced disintegration. First, TPGS acts as a non-ionic surfactant that drastically lowers the solid–liquid interfacial tension, transforming the hydrophobic surface of crystalline TPZ into a highly hydrophilic interface [28]. In addition, at concentrations exceeding its critical micelle concentration, TPGS forms self-assembled micelles that entrap unionized TPZ molecules within their hydrophobic core, preventing recrystallization and boosting apparent aqueous solubility in neutral media [28]. Moreover, MCC functions as a porous carrier that rapidly draws the dissolution medium into the granule interior via capillary action, promoting immediate granule disintegration and preventing particle agglomeration during dissolution [32,33].

### 2.4. Morphological and Physical Characteristics of TPZ MG

The surface morphology and structural features of intact TPZ raw material and the TPZ MG formulation were evaluated using SEM (Figure 4a,b).

SEM observations showed that the TPZ raw material consisted of discrete, relatively spherical crystalline particles with clear geometric boundaries, ranging from 50 µm to 200 µm (Figure 4a). In contrast, the TPZ MG formulation exhibited a distinctly different morphology, forming irregular, aggregated structures with rougher surfaces and larger sizes spanning 200 µm to 500 µm (Figure 4b). The individual crystal boundaries of TPZ particles were not distinguishable in the granule. This morphological transformation indicates that during the MG process, molten TPGS uniformly coated and bound the MCC particles together with TPZ particles, constructing a consolidated composite solid matrix wherein TPZ particles were physically embedded and dispersed within the TPGS/MCC carrier phase [13,18].

The solid-state characterization and crystalline state of TPZ within the matrix were investigated via PXRD and DSC (Figure 4c,d). Intact TPZ exhibited sharp, intense crystalline reflections at 2*θ* = 12.5°, 14.9–17.5°, 19.5°, 22.8° and 24.4°, verifying its crystalline form (Figure 4c). Although characteristic TPZ peaks at 2θ = 19.5°, 22.8°, and 24.4° were masked by overlapping diffraction signals from the TPGS/MCC vehicle matrix, particularly the characteristic TPGS reflections observed at approximately 19° and 23° [38], representative TPZ reflections at 2θ = 12.5°, 15.1°, 15.7°, 16.7°, and 17.5° were preserved at identical 2θ angles in the TPZ MG formulation. The data support that the enhanced dissolution of TPZ via the MG system is not attributed to complete drug amorphization, as PXRD confirmed the retention of drug crystallinity. The observed decrease in peak intensity stems from both a mass-dilution effect induced by TPGS and MCC and partial thermal solubilization of TPZ in molten TPGS, which forms a molecularly dispersed fraction. In our preliminary study, the equilibrium solubility of TPZ in TPGS at the processing temperature of 70 °C was approximately 5–10 mg/mL. Consequently, out of the total 50 mg drug loading per formulation unit, approximately 5–10 mg of TPZ was dissolved in molten TPGS at elevated processing temperature and molecularly dispersed in an amorphous state upon cooling, while the remaining 40–45 mg remained suspended and dispersed as a stable crystalline form within the TPGS/MCC solid matrix.

In the DSC analysis, intact TPZ raw material exhibited a sharp, well-defined endothermic melting peak at approximately 225 °C, corresponding to its intrinsic T_m_ (Figure 4d). In contrast, this drug melting peak disappeared in the TPZ MG formulation. Instead, the thermogram of TPZ MG closely mirrored that of the blank vehicle, displaying two distinct thermal transitions: a sharp low-temperature endothermic peak at around 38–40 °C and a broad high-temperature endothermic peak spanning 300–350 °C. The first peak corresponds to the melting transition (T_m_) of TPGS [39], whereas the second broad peak is attributed to the thermal decomposition and degradation of MCC [40]. This disappearance of the drug melting peak is a well-known phenomenon in surfactant-rich systems [39]: as the DSC temperature rises, TPGS melts first (<40 °C) and acts as a liquid solvent that dissolves the crystalline TPZ before it reaches 225 °C. Taken together, the PXRD and DSC findings confirm that TPZ exists predominantly in a crystalline form distributed within the TPGS/MCC matrix, accompanied by a small molecularly dispersed fraction. This unique structural setup ensures both physical stability during storage and superior dissolution kinetics.

### 2.5. Physicochemical Stability of TPZ MG

The physicochemical stability of the finalized TPZ MG was evaluated under long-term (25 °C/60% RH) and accelerated conditions (40 °C/75% RH) for 24 weeks. Drug content and degradation products (related substances) were monitored throughout the 24-week period, while crystalline state and in vitro dissolution behavior were evaluated at the end of the 24-week storage period. Physicochemical stability testing is a critical quality attribute in pharmaceutical formulation development, particularly for lipid- or polymer-based solid dispersion systems [12]. Such formulations frequently encounter physical instability issues during storage, wherein metastable amorphous drug fractions undergo phase separation, moisture absorption, or progressive recrystallization into lower-energy polymorphic states, leading to changes in dissolution and even chemical degradation [12,41].

The finalized TPZ MG exhibited excellent chemical and physical stability throughout the 24-week evaluation period. The drug content remained highly stable, with an initial value of 108.97 ± 0.54% and values of 101.62 ± 1.83% and 102.90 ± 0.60% at 24 weeks for the samples stored under long-term and accelerated conditions, respectively, with no marked alterations (Figure 5a).

Furthermore, the level of related substances was maintained near the baseline value of 0.425 ± 0.044% throughout the 24 weeks, reaching 0.443 ± 0.068% and 0.487 ± 0.003%, respectively, at 24 weeks, remaining strictly below 0.6% without any significant accumulation (Figure 5b). Previous forced-degradation studies revealed that TPZ is susceptible to distinct degradation pathways depending on stress conditions [36]. Under acidic and oxidative conditions, ether bond cleavage of TPZ yields 4-hydroxy-*N*,*N*,2-trimethyl-1*H*-benzo[d]imidazole-6-carboxamide and 5,7-difluorochroman-4-ol. Conversely, exposure to basic conditions induces amide hydrolysis, forming *(S)*-4-((5,7-difluorochroman-4-yl)oxy)-2-methyl-1*H*-benzo[d]imidazole-6-carboxylic acid. Furthermore, oxidative stress targets five vulnerable positions within the TPZ structure—specifically, all three nitrogen atoms as well as the C-2 and C-3 positions on the chroman ring. Despite these known degradation liabilities, the TPZ MG showed no appreciable chemical degradation during storage, as evidenced by the absence of marked accumulation of related substances. This stability is largely attributed to the fact that a substantial fraction of TPZ remains in its thermodynamically stable crystalline state dispersed within the solid matrix, coupled with the protective microenvironment formed by TPGS and MCC.

Solid-state characterization via PXRD further confirmed that the characteristic 2*θ* Bragg diffraction peaks (e.g., 12.7°, 15.1°, 15.7°, 16.7°, and 17.3°) of TPZ in the MG remained unchanged in position compared to the initial state (Figure 5c). In parallel, in vitro dissolution testing in simulated intestinal fluid (pH 6.8 phosphate buffer) yielded dissolution profiles virtually identical to the initial release pattern (*f*_2_ > 50; specifically, *f*_2_ = 93.7 and 68.8 for the 24-week samples stored under long-term and accelerated conditions, respectively), demonstrating no significant retardation or alteration in dissolution kinetics after 24 weeks (Figure 5d). Taken together, we concluded that the TPZ MG was physicochemically stable throughout the 24-week storage period, ensuring a reliable shelf life without compromising pharmaceutical quality.

### 2.6. In Vivo PK Profile of TPZ MG in Rats

TPZ is characterized by high membrane permeability but severely restricted pH-dependent aqueous solubility (<0.05 mg/mL at neutral pH) [8]. Following oral administration, the primary site of systemic absorption for TPZ is reported to be the upper small intestine (duodenum and jejunum) [3,7]. However, as the drug moves from the acidic environment of the stomach to the higher pH conditions of the intestinal lumen, extensive deprotonation drastically reduces its baseline solubility, triggering erratic drug precipitation, low dissolution rates, and substantial intra- and inter-individual pharmacokinetic variability [8,10].

In this study, the PK profile of TPZ MG was comparatively evaluated in Sprague–Dawley rats with drug powder (unformulated API) following a single oral dose (10 mg/kg) [9]. The resulting plasma concentration–time profiles and key PK parameters are presented in Figure 6 and Table 2, respectively.

Following oral administration, TPZ MG exhibited higher plasma concentration levels and significantly increased systemic exposure compared to TPZ raw material. Furthermore, a distinct acceleration in absorption kinetics was observed for the MG formulation, where the time required to reach peak plasma concentration (T_max_) was substantially shortened from 2.25 ± 1.17 h for the unformulated API to 0.65 ± 0.34 h for TPZ MG. This rapid onset of absorption aligns closely with the rapid initial dissolution kinetics observed in vitro at early time points (within 5–10 min). As detailed in Table 2, C_max_ of TPZ raw material was 274.72 ± 86.14 ng/mL, whereas the TPZ MG achieved a markedly elevated C_max_ of 669.69 ± 171.45 ng/mL—representing an approximate 2.44-fold increase. Similarly, AUC_0–12 h_ increased from 715.40 ± 101.19 ng·h/mL for the drug raw material to 1305.06 ± 187.69 ng·h/mL for the MG formulation, demonstrating a 1.82-fold enhancement in total systemic exposure. Meanwhile, the t_1/2_ remained comparable between the two groups (2.08 ± 0.43 h for raw material vs. 1.88 ± 0.15 h for MG). When compared to previously reported human pharmacokinetic data [1], these preclinical results demonstrated consistent pharmacokinetic behavior in terms of absorption rate and elimination profiles. In human clinical studies, orally administered TPZ typically exhibits a rapid absorption pattern (T_max_ ranging around 0.5–1.5 h) and a relatively short elimination half-life (t_1/2_ ~ 3.65–5.39 h). The T_max_ observed in our preclinical model was consistent with the rapid absorption reported in humans, whereas the shorter elimination half-life likely reflects interspecies differences in drug disposition.

The observed in vivo PK superiority of TPZ MG strongly correlates with the in vitro dissolution behavior demonstrated across various media (Figure 3). In neutral and weakly acidic media mimicking the intestinal lumen (pH 6.8 and DW), the unformulated TPZ API exhibited severely restricted dissolution rates (<35% at 120 min) due to its poor aqueous solubility (<0.05 mg/mL) and hydrophobic surface nature, making crystal dissolution the ultimate rate-limiting step for oral absorption [8]. Conversely, in TPZ MG, this thermodynamic absorption barrier was overcome through a synergistic combination of surfactant-driven interfacial wetting and micellar solubilization by TPGS, capillary wicking and rapid disintegration mediated by the porous MCC matrix, and an expanded effective surface area achieved by uniform physical dispersion of crystalline TPZ particles within the carrier phase [28,33]. Previous studies on polymeric ASDs of TPZ reported that dissolution enhancement led to a 1.5- to 2.1-fold increase in C_max_ and AUC in rodent models [9,11]. Specifically, Won et al. fabricated TPZ ASDs using HPMC and PVP polymers, reporting a 2.1-fold improvement in oral absorption [9]. However, conventional ASD techniques often require organic solvents—such as ethanol, methanol, or dichloromethane—during solvent evaporation or spray drying processes, alongside high polymer-to-drug ratios to prevent thermodynamic recrystallization during processing and storage. In contrast, the present TPZ MG system resulted in a 1.82-fold increase in AUC_0–12 h_ and a 1.78-fold increase in AUC_0–∞_ compared with the raw API, together with a 2.44-fold increase in C_max_, despite being prepared through a solvent-free hot-melt process. Taken together, these findings suggest that the crystalline drug-loaded TPGS/MCC-based MG system can be a highly effective approach for improving the oral absorption of TPZ while providing robust physicochemical stability.

## 3. Materials and Methods

### 3.1. Materials

TPZ drug powder (purity > 98.5%, mean particle size of 78.7 µm) was kindly provided by Tianjin Propharm Co., Ltd. (Lot No. 2201001, Tianjin, China). Tegoprazan-d_6_ was purchased from MedChemExpress (MCE; Monmouth Junction, NJ, USA). TPGS was purchased from PMC Isochem (Vert-le-Petit, France). Gelucire 44/14 (lauroyl polyoxyl-32 glycerides) and Gelucire 50/13 (stearoyl polyoxyl-32 glycerides) were obtained from Gattefossé (Saint-Priest, France). Polyoxyl 40 hydrogenated castor oil (Kolliphor RH 40), Poloxamer 188, and Poloxamer 407 were acquired from BASF (Ludwigshafen, Germany). Microcrystalline cellulose (MCC; Avicel PH-102, average molecular weight (MW) ~ 36,000 g/mol) was purchased from IFF Pharma Solutions (Wilmington, DE, USA). Hydroxypropyl methylcellulose (HPMC; Hypromellose 2910, E50 grade, MW ~ 10,000 g/mol) was supplied by Colorcon (Harleysville, PA, USA). Polyvinylpyrrolidone K17 (PVP K17; MW ~ 10,000 g/mol) was obtained from CTC Bio Inc. (Hwaseong, Republic of Korea). Sodium carboxymethyl cellulose (Na-CMC; low viscosity grade, MW ~ 90,000 g/mol) was purchased from Sigma-Aldrich (St. Louis, MO, USA). Colloidal silicon dioxide (Aerosil 200 Pharma) was supplied by Evonik Industries AG (Essen, Germany). High-performance liquid chromatography (HPLC)-grade acetonitrile was purchased from Samchun Pure Chemical Co., Ltd. (Gyeonggi-do, Republic of Korea). HPLC-grade acetonitrile and water for liquid chromatography-tandem mass spectrometry (LC–MS/MS) analysis were purchased from Tedia (Fairfield, OH, USA). HPLC-grade ethyl acetate was purchased from J. T. Baker (Radnor, PA, USA). All other chemical reagents and solvents were of analytical or HPLC grade and used as received without further purification.

### 3.2. Preparation of TPZ-Loaded MGs

TPZ-loaded MGs were prepared using a solvent-free hot-melt method [18]. Briefly, a selected solid carrier or surfactant—evaluated among Gelucire 44/14, Gelucire 50/13, TPGS, Kolliphor RH 40, Poloxamer 188, and Poloxamer 407—was placed in a 20 mL scintillation vial and fully melted at 70 °C in a water bath. TPZ was then added to the molten carrier under continuous, thorough mixing for 1 h to ensure uniform drug dispersion. Subsequently, secondary solid carriers (MCC, HPMC E50, PVP K17, Na-CMC, or Aerosil 200) were incorporated into the molten mixture at 70 °C and blended until a homogeneous mass was achieved, maintaining a fixed TPZ:TPGS:carrier ratio of 1:2:2 (*w*/*w*/*w*). To optimize formulation composition, the weight ratios of TPZ:TPGS (ranging from 1:1 to 1:4 *w*/*w*) and MCC content were varied to yield final TPZ:TPGS:MCC ratios of 1:1:1, 1:2:2, 1:2:4, 1:2:6, 1:3:3, and 1:4:4 (*w*/*w*/*w*), respectively. After mixing was completed, the molten formulation was solidified by cooling at −20 °C for 1 h. The resulting solid mass was harvested, pulverized, and stored at room temperature (25 ± 2 °C) for further physicochemical characterization.

### 3.3. HPLC Analysis for Drug Content and Degradation Products

The drug content and degradation products of TPZ in MGs were analyzed using an Agilent 1200 Infinity HPLC system (Agilent Technologies, Santa Clara, CA, USA) equipped with a binary pump (G1212B), column oven (G1316B), UV/Vis detector (G1314B), and autosampler (G1367B). The HPLC conditions were adapted from a previously reported method for TPZ with minor modifications [10]. To determine the drug content in the formulation, an amount of formulation equivalent to 2 mg of TPZ was transferred into a 10 mL volumetric flask and diluted to volume with acetonitrile. The mixture was completely dissolved via sonication. To remove insoluble excipients, the sample was centrifuged at 13,000 rpm and 20 °C for 10 min. The resulting supernatant was collected, diluted 2-fold with the mobile phase, and injected into the HPLC system. For the quantification of TPZ, chromatographic separation was achieved using a reverse-phase INNO C18 column (4.6 mm I.D. × 150 mm, 3 µm; Youngjin Biochrom, Seongnam, Republic of Korea) maintained at 30 °C. The mobile phase consisted of a 3:7 (*v*/*v*) mixture of 0.01 M ammonium acetate and acetonitrile, operated in isocratic mode at a flow rate of 1.0 mL/min. The injection volume was 20 µL, and UV detection was performed at a wavelength of 220 nm. Under these conditions, the retention time of TPZ was approximately 2.1 min. The calibration curve exhibited excellent linearity over the concentration range of 1–50 µg/mL (y = 136.04x + 25.829, r^2^ = 1.0000). The limit of detection (LOD) and limit of quantitation (LOQ) were determined to be 0.05 µg/mL and 0.16 µg/mL, respectively.

For the analysis of related substances, a gradient elution method was employed using the same HPLC system and column. Mobile phase A consisted of 0.01 M ammonium acetate and acetonitrile (19:1, *v*/*v*), and mobile phase B was 100% acetonitrile. The gradient program was set as follows: 0–2 min, 12% B; 2–3 min, linear increase to 30% B; 3–15 min, hold at 30% B; 15–16 min, linear increase to 80% B; 16–18 min, linear increase to 90% B; 18–20 min, hold at 90% B; 20–20.01 min, return to initial conditions (12% B); and 20.01–25 min, re-equilibration with 12% B. The flow rate was maintained at 0.8 mL/min, with an injection volume of 20 µL and a column temperature of 30 °C.

### 3.4. Morphological and Physical Characterization of TPZ MGs

#### 3.4.1. SEM Observations

The surface morphology of raw materials, physical mixtures, and optimized TPZ MG formulations was evaluated using an SEM (JSM-6510, JEOL, Tokyo, Japan). Individual powder samples were mounted onto aluminum stubs fitted with double-sided conductive carbon tape. To prevent electrostatic charging during electron beam irradiation, the mounted samples were sputter-coated with a thin layer of gold using an automated sputter coater (Model 108AUTO, Cressington Scientific Instruments, Watford, UK) under a vacuum atmosphere. Morphological observations were conducted at an accelerating voltage of 15 kV.

#### 3.4.2. PXRD Analysis

The crystalline state of raw TPZ, individual excipients, physical mixtures, and TPZ MGs was determined using a high-resolution X-ray diffractometer (Ultima IV, Rigaku Corporation, Tokyo, Japan) equipped with Cu-Kα radiation (λ = 1.5418 Å). The samples were packed evenly into standard glass sample holders to ensure a flat analysis surface. The PXRD patterns were collected in the 2*θ* scanning range from 5° to 40° in continuous scan mode with a step size of 0.02° and a scanning speed of 2°/min. The generator voltage and current were maintained at 40 kV and 40 mA, respectively.

#### 3.4.3. DSC Analysis

The thermal behavior of raw TPZ, physical mixtures, and TPZ MG was analyzed using a DSC (Thermo Plus EVO2, Rigaku Corporation, Tokyo, Japan). Accurately weighed samples (approximately 1–2 mg) were sealed in standard aluminum pans with pinholed lids. An empty aluminum pan was used as a reference. The thermal profiles were recorded by heating the samples from 0 °C to 400 °C at a constant heating rate of 10 °C/min under a continuous nitrogen gas purge to maintain an inert atmosphere. The T_m_ was evaluated from the resulting thermograms.

### 3.5. In Vitro Dissolution Profile of TPZ from MGs

The in vitro dissolution profiles of raw TPZ, commercial film-coated tablet (K-CAB Tab. 50 mg, batch No. AA526F0; HK inno.N Corporation, Cheongju, Republic of Korea) and different TPZ MG formulations were evaluated using a USP Apparatus II (paddle method; DT 126, Erweka, Heusenstamm, Germany) [10]. The dissolution study was performed in 900 mL of four distinct media representing physiological pH conditions: simulated gastric fluid (pH 1.2), acetate buffer (pH 4.0), phosphate buffer (pH 6.8), and DW. Formulations containing an amount equivalent to 50 mg of TPZ were introduced into each dissolution vessel. The dissolution medium was maintained at 37 ± 0.5 °C and stirred at a paddle speed of 50 rpm. At predetermined time intervals (0.083, 0.167, 0.25, 0.5, 1, and 2 h), a 1.0 mL aliquot of the dissolution medium was withdrawn and immediately replaced with an equal volume of fresh, pre-warmed (37 °C) medium to maintain a constant dissolution volume. The collected samples were centrifuged at 13,000 rpm and 20 °C for 10 min to remove insoluble materials. Thereafter, a 500 µL aliquot of the resulting supernatant was diluted 2-fold with an equal volume of the mobile phase mixture (0.01 M ammonium acetate:acetonitrile = 3:7, *v*/*v*). The diluted samples were quantified using the HPLC analytical method described in Section 3.3. To compare the dissolution profiles between the initial formulation and the stored stability samples, the similarity factor (*f*_2_) was calculated using the following equation [42,43]:

(1) $$f_{2}=50\times \log_{10}\left[{\left(1+\frac{1}{n}\sum_{t=1}^{n}{\left(R_{t}-T_{t}\right)}^{2}\right)}^{-0.5}\times 100\right],$$

where *n* is the number of time points, *R_t_* is the mean percentage dissolved of the initial formulation at time point *t*, and *T_t_* is the mean percentage dissolved of the stored stability formulation at time point *t*. An *f*_2_ value between 50 and 100 indicates equivalence or similarity between the two dissolution profiles (with a difference of less than 5–10% across all time points).

### 3.6. Storage Stability of TPZ MGs

The physicochemical stability of the TPZ MG formulation was evaluated under long-term (25 ± 2 °C/60 ± 5% RH) and accelerated (40 ± 2 °C/75 ± 5% RH) storage conditions for 24 weeks. TPZ MG formulation samples (equivalent to 500 mg) were weighed into 20 mL clear glass scintillation vials, tightly sealed with screw caps, and wrapped in aluminum foil for light protection. The samples were withdrawn at predetermined time intervals of 1, 2, 4, 8, 12, and 24 weeks. Drug content (%) and the levels of individual and total related substances (%) were quantitatively analyzed at each sampling time point using the HPLC method described in Section 3.3. At the end of the 24-week storage period, the in vitro dissolution profiles and crystalline state of TPZ in the MGs were further evaluated using the methods described in Section 3.5 and Section 3.4.2, respectively.

### 3.7. In Vivo Pharmacokinetic Profile of TPZ Following Oral Administration of MGs

#### 3.7.1. Animals and Experimental Design

All animal procedures were reviewed and approved by the Institutional Animal Care and Use Committee (IACUC) of Dankook University (Approval No. DKU-25-044; approved on 13 June 2025). Male Sprague–Dawley rats (6–9 weeks old, weighing 250 ± 20 g) were purchased from Samtako Bio Korea (Gyeonggi-do, Republic of Korea). The animals were housed under controlled environmental conditions (23 ± 1 °C, 50 ± 5% relative humidity) with a 12 h light/dark cycle and were provided free access to food and water. Prior to the experiments, all rats were acclimated for 3 days and subsequently fasted for 12 h with free access to water. The rats were randomly divided into two groups (*n* = 5 per group): (1) raw TPZ powder and (2) optimized TPZ MG formulation. Based on preliminary studies, the oral dose was set at 10 mg/kg of TPZ [9], administered at a fixed volume of 1.0 mL/kg (0.25 mL per rat). Raw TPZ powder was suspended in 0.5% (*w*/*v*) methylcellulose solution, whereas the TPZ MG formulation was dispersed in DW immediately prior to administration. Both formulations were orally administered using a gavage needle (Ø 1.18 × 75 mm). Blood samples (approximately 0.5 mL) were collected from the jugular vein into heparinized (50 IU) syringes using a 26-gauge needle at predetermined time points: 0.25, 0.5, 0.75, 1, 1.5, 2, 4, 6, 8, and 12 h post-administration. The collected blood samples were immediately centrifuged at 13,000 rpm and 4 °C for 10 min to harvest plasma. The obtained plasma samples were stored in a deep freezer at −80 °C until LC–MS/MS analysis.

#### 3.7.2. LC–MS/MS Analysis of TPZ in Rat Plasma

The level of TPZ in rat plasma was quantified using a validated LC–MS/MS method [9,44]. Plasma samples (50 µL) were processed via liquid–liquid extraction; a 50 µL aliquot of water containing an internal standard (IS; Tegoprazan-d_6_ 50 ng/mL in water) and a 400 µL aliquot of ethyl acetate were added to each sample. The mixture was vortexed thoroughly for 5 min and centrifuged at 13,000 rpm for 5 min. The supernatant was transferred to a clean tube and evaporated under a gentle stream of nitrogen gas. The residue was reconstituted with 150 µL of mobile phase. A 2 µL aliquot was directly injected into the LC–MS/MS system.

Chromatographic separation was performed on an Agilent Infinity 1260 HPLC system (Agilent Technologies, Santa Clara, CA, USA) equipped with a Synergi Polar RP column (4 µm particle size, 2.0 mm × 150 mm; Phenomenex, Torrance, CA, USA). The mobile phase consisted of 0.1% (*v*/*v*) formic acid in water and 0.1% (*v*/*v*) formic acid in acetonitrile (20:80, *v*/*v*), delivered at a flow rate of 0.25 mL/min under an optimized isocratic elution program. Mass spectrometric detection was achieved using an Agilent 6470 Triple Quadrupole mass spectrometer equipped with an electrospray ionization (ESI) source operated in the positive ion mode. Quantification was performed using multiple reaction monitoring (MRM) mode with the transitions *m*/*z* 388.3 → 220.2 for TPZ and *m*/*z* 394.3 → 226.2 for the IS. Data acquisition and processing were performed using MassHunter software (Version B.08.00; Agilent Technologies).

#### 3.7.3. Pharmacokinetic Data Analysis

Pharmacokinetic parameters were determined via non-compartmental analysis (NCA) using WinNonlin software (Version 8.1; Pharsight Corp., Mountain View, CA, USA). The maximum plasma concentration (C_max_) and the time to reach C_max_ were obtained directly from the observed plasma concentration–time profiles. The area under the plasma concentration–time curve from zero to the last measurable time point (AUC_0–12 h_) was calculated using the linear trapezoidal rule. The area under the curve extrapolated to infinity (AUC_0–∞_) was calculated as AUC_0–*t*_ + *C_t_*/*λ_z_*, where C*_t_* is the last observed quantifiable concentration and *λ_z_* is the terminal elimination rate constant. The terminal elimination half-life (t_1/2_) was determined as ln(2)/*λ_z_*, where *λ_z_* was estimated from the slope of the linear regression of the log-linear terminal phase of the plasma concentration–time data.

### 3.8. Statistical Analysis

All experimental data are expressed as the mean ± SD of at least three independent measurements (*n* ≥ 3). Pharmacokinetic data are presented as the mean ± SE. Statistical differences in C_max_, AUC_0–12 h_, AUC_0–∞_, and t_1/2_ between the two pharmacokinetic groups were evaluated using Welch’s unpaired *t*-test, whereas T_max_ was analyzed using the Mann–Whitney test. A *p*-value of less than 0.05 (*p* < 0.05) was considered statistically significant.

## 4. Conclusions

In the present study, a novel TPGS/MCC-based MG system was successfully formulated to improve the oral bioavailability of TPZ. By utilizing a solvent-free hot-melt process, TPZ was uniformly incorporated in its crystalline state into a solid matrix composed of TPGS and MCC at an optimized weight ratio of 1:2:4. Comprehensive solid-state characterizations revealed that TPZ predominated in its thermodynamically stable crystalline form within the carrier matrix, preventing processing- and storage-induced physical phase transitions. The TPZ MG exhibited marked dissolution enhancement across various media (pH 1.2, 4.0, 6.8, and DW) through a synergistic mechanism involving surfactant-mediated wetting, micellar solubilization, and capillary wicking-driven disintegration. Furthermore, in the storage stability study, TPZ MG samples were maintained under the designated storage conditions (25 °C/60% RH and 40 °C/75% RH) for 24 weeks. The MG system maintained its physicochemical stability over the 24-week study period, with no marked alterations in drug content, related substances, crystalline state, or dissolution profiles. In a pharmacokinetic study in rats, the optimized TPZ MG achieved significant enhancement in systemic exposure, displaying a 2.44-fold increase in C_max_ and a 1.82-fold increase in AUC_0–12 h_ alongside accelerated absorption kinetics (T_max_ = 0.65 h) relative to raw TPZ powder. Taken together, these findings indicate that crystalline drug-loaded MG represents a simple and efficient platform technology to enhance the oral bioavailability of poorly water-soluble drugs like TPZ.

## Figures and Tables

**Figure 1:**
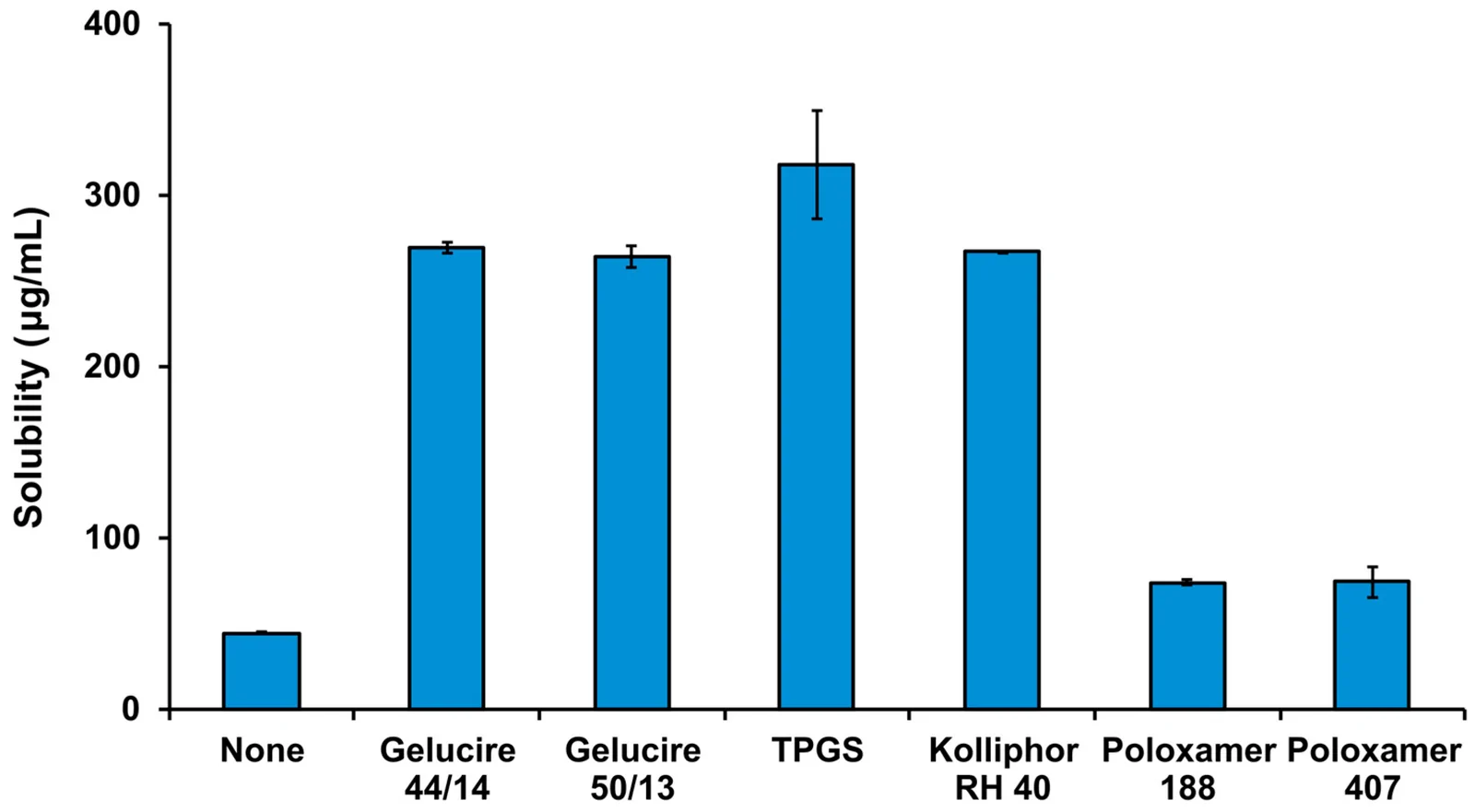
Equilibrium solubility of TPZ in phosphate buffer (10 mM, pH 6.8) containing various solid surfactants. Each surfactant was incorporated into the aqueous medium at a concentration of 1% (*w*/*v*). Data are expressed as mean ± standard deviation (SD) (*n* = 3).

**Figure 2:**
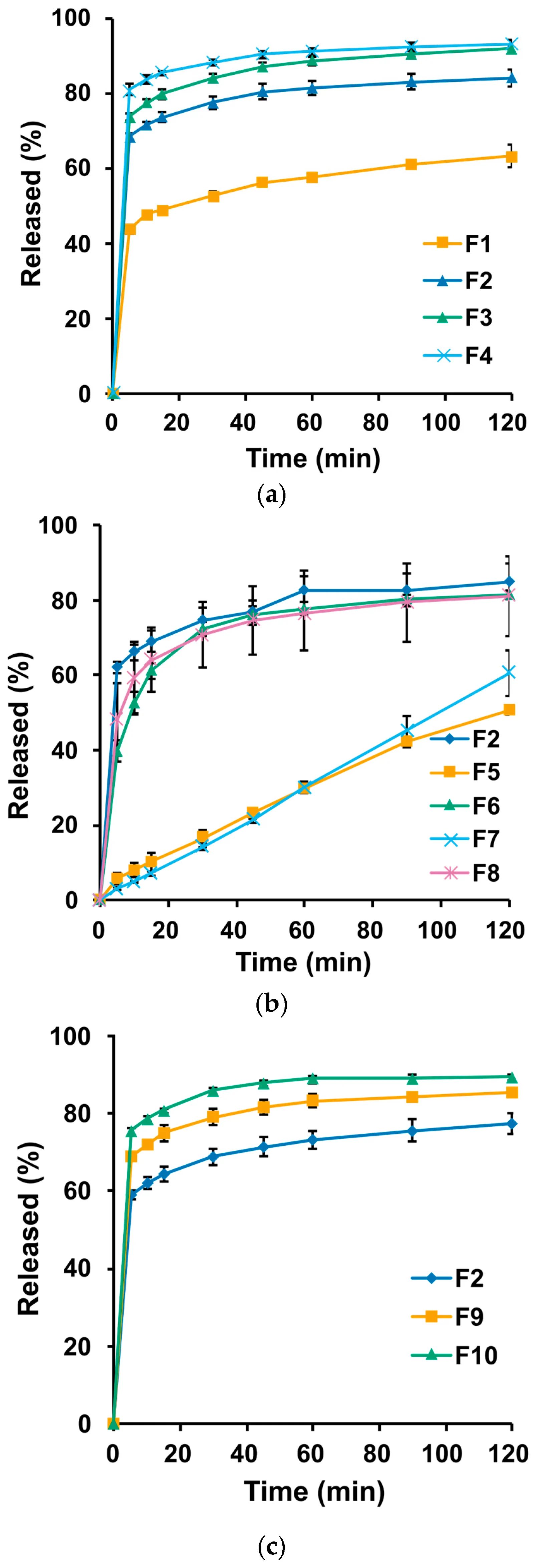
In vitro dissolution profiles of TPZ-loaded MGs in pH 6.8 phosphate buffer as a function of (**a**) drug-to-TPGS ratio (F1–F4), (**b**) carrier types (F2 and F5–F8), and (**c**) MCC amount (F2, F9, and F10) (USP Apparatus II, 50 rpm). Data are presented as mean ± SD (*n* = 3).

**Figure 3:**
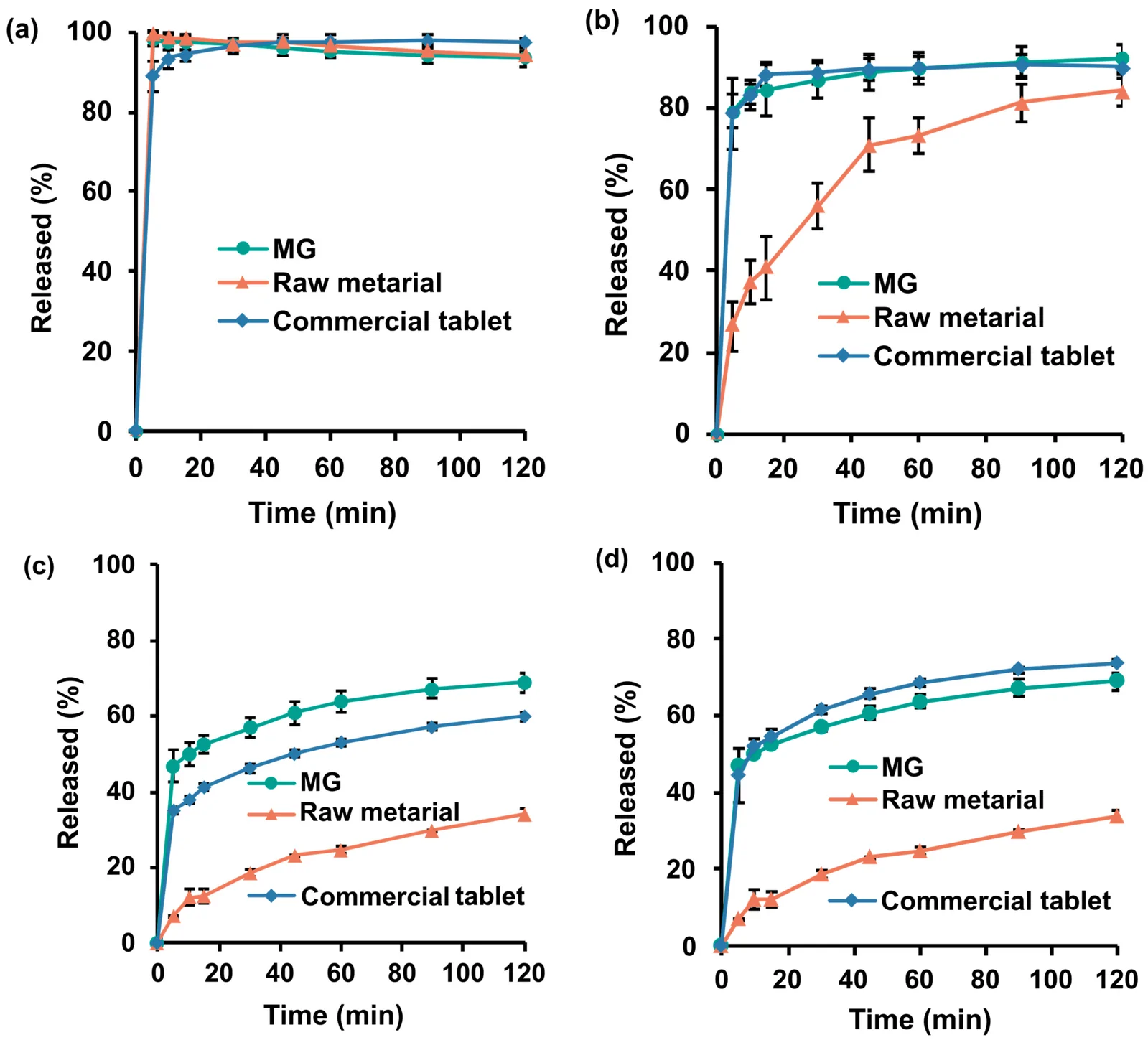
In vitro dissolution profiles of intact TPZ raw material, commercial film-coated tablet, and TPZ MG across various dissolution media: (**a**) pH 1.2, (**b**) pH 4.0, (**c**) pH 6.8, and (**d**) DW (USP Apparatus II, 50 rpm). Data are presented as mean ± SD (*n* = 3).

**Figure 4:**
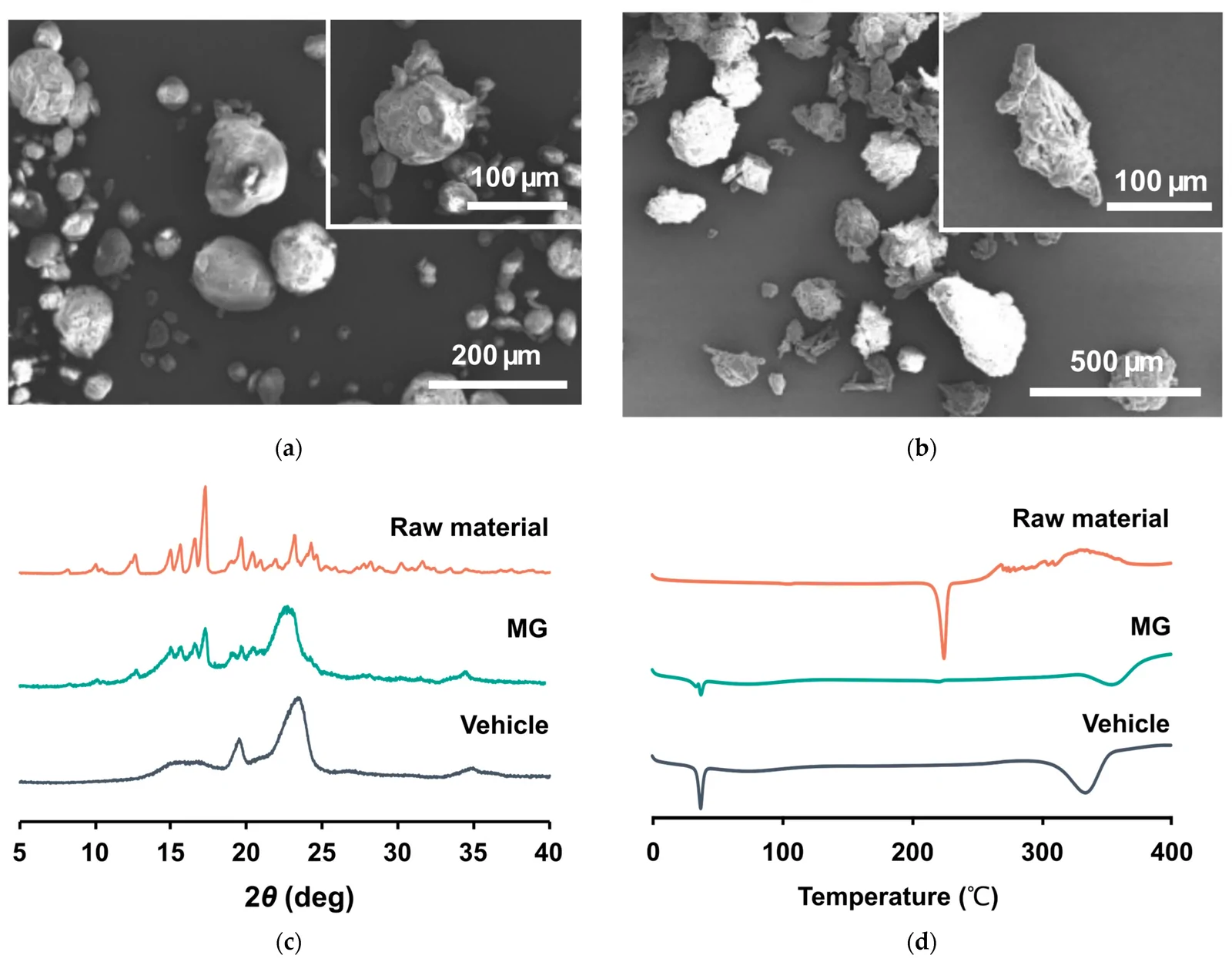
Morphological and physical characteristics of the TPZ MG. Representative SEM images of (**a**) intact TPZ raw material and (**b**) TPZ MG. (**c**) PXRD patterns and (**d**) DSC thermograms of intact TPZ raw material, vehicle, and TPZ MG. Vehicle represents a blank carrier matrix without TPZ.

**Figure 5:**
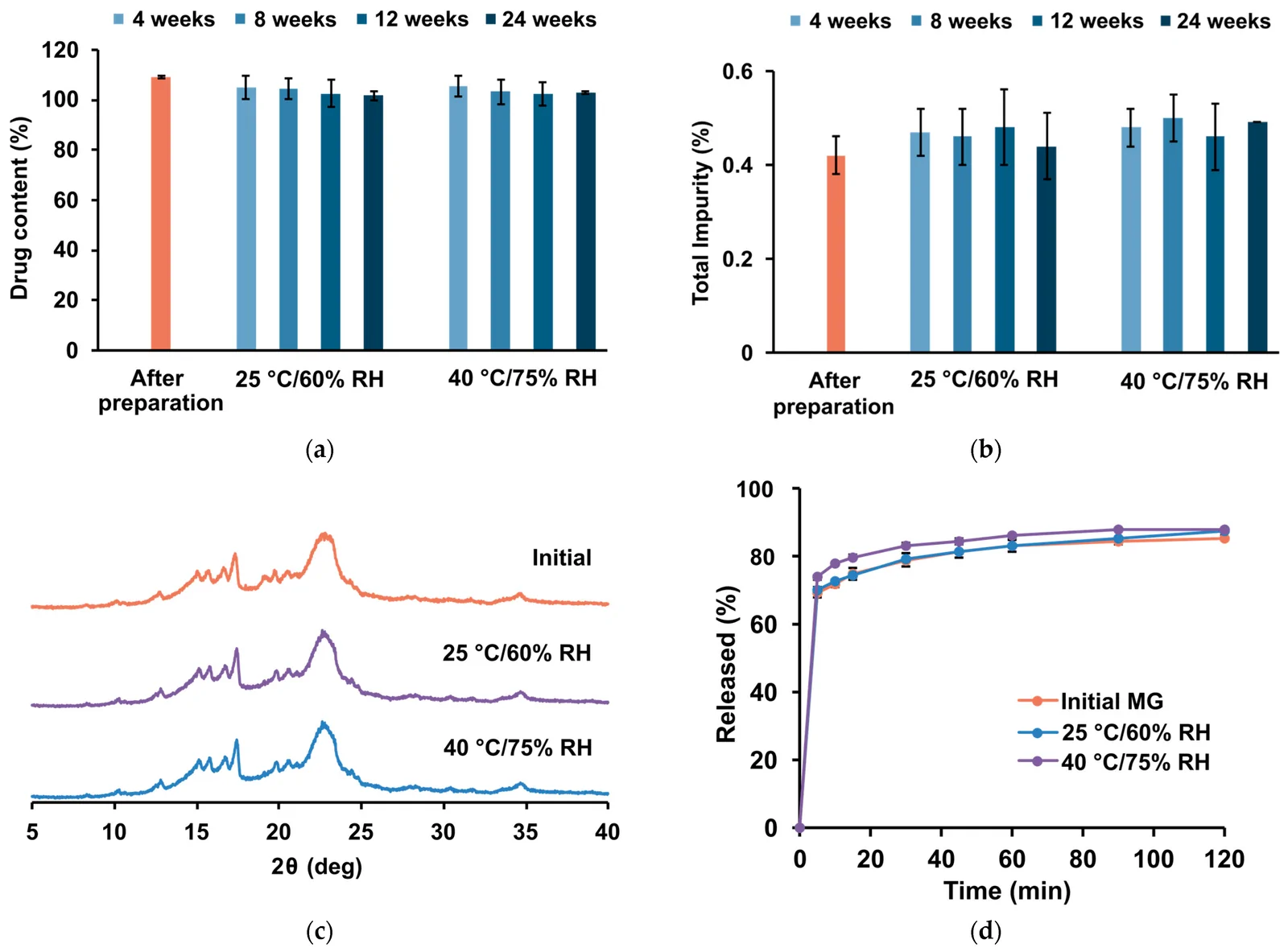
Physicochemical stability of TPZ MG following 24 weeks of storage at 25 °C/60% RH or 40 °C/75% RH. (**a**) Drug content, (**b**) related substances, (**c**) PXRD patterns, and (**d**) in vitro dissolution profiles of TPZ MG in pH 6.8 phosphate buffer. Drug content, related substances, and dissolution data are presented as mean ± SD (*n* = 3); PXRD patterns are representative profiles.

**Figure 6:**
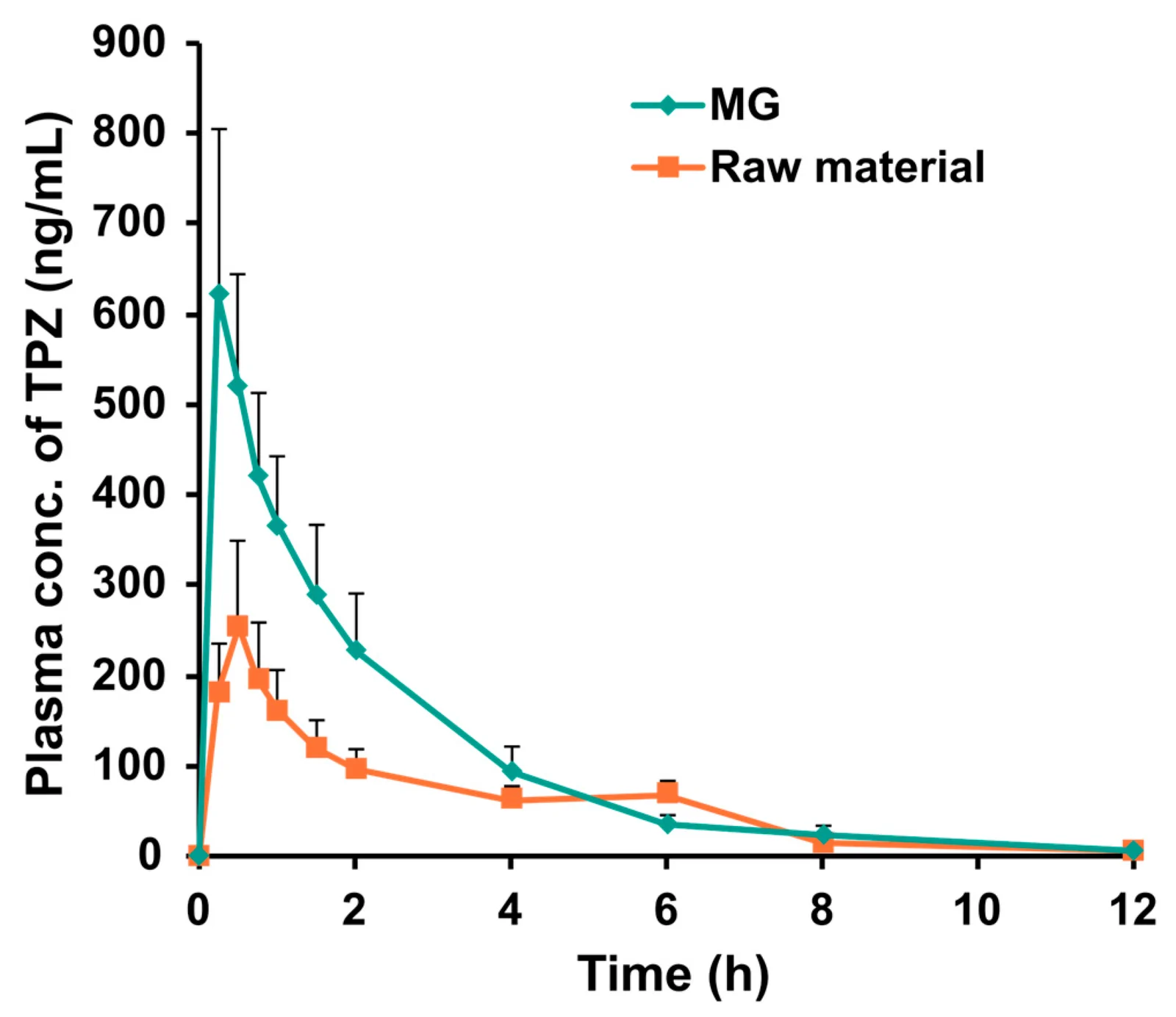
Plasma concentration–time profiles of TPZ following a single oral administration of TPZ raw material and TPZ MG in Sprague–Dawley rats (10 mg/kg as TPZ). Unformulated TPZ raw material was suspended in 0.5% (*w*/*v*) methylcellulose, whereas TPZ MG was dispersed in DW prior to dosing.

**Table 1:** Compositions of TPGS-based TPZ MG formulations.

Ingredients	F1	F2	F3	F4	F5	F6	F7	F8	F9	F10
TPZ (mg)	50	50	50	50	50	50	50	50	50	50
TPGS (mg)	50	100	150	200	100	100	100	100	100	100
Carrier type	MCC	MCC	MCC	MCC	HPMC E50	PVP K17	Na-CMC	Aerosil 200	MCC	MCC
Carrier amount (mg)	50	100	150	200	100	100	100	100	200	300

**Table 2:** PK parameters of TPZ following a single oral administration of TPZ raw material and TPZ MG in Sprague–Dawley rats. Data are presented as mean ± standard error (SE) (*n* = 5). C_max_, AUC_0–12 h_, AUC_0–∞_, and t_1/2_ were compared using Welch’s unpaired *t*-test, whereas T_max_ was analyzed using the Mann–Whitney test. *p* < 0.05 was considered statistically significant. * *p* < 0.05 vs. Raw material.

Parameters	Raw Material	MG
C_max_ (ng/mL)	274.72 ± 86.14	669.69 ± 171.45
AUC_0–12 h_ (ng·h/mL)	715.40 ± 101.19	1305.06 ± 187.69 *
AUC_0–∞_ (ng·h/mL)	744.50 ± 93.16	1325.83 ± 188.54 *
T_max_ (h)	2.25 ± 1.17	0.65 ± 0.34
t_1/2_ (h)	2.08 ± 0.43	1.88 ± 0.15

## Data Availability

Data is contained within the article.

## References

[1] Han S., Choi H.Y., Kim Y.H., Nam J.Y., Kim B., Song G.S., Lim H.S., Bae K.S. (2019). Randomised clinical trial: Safety, tolerability, pharmacokinetics, and pharmacodynamics of single and multiple oral doses of tegoprazan (CJ-12420), a novel potassium-competitive acid blocker, in healthy male subjects. Aliment. Pharmacol. Ther..

[2] Takahashi N., Take Y. (2018). Tegoprazan, a Novel Potassium-Competitive Acid Blocker to Control Gastric Acid Secretion and Motility. J. Pharmacol. Exp. Ther..

[3] Yoon D.Y., Sunwoo J., Shin N., Kim A.R., Kim B., Song G.S., Jang I.J., Lee S. (2021). Effect of meal timing on pharmacokinetics and pharmacodynamics of tegoprazan in healthy male volunteers. Clin. Transl. Sci..

[4] Han S., Choi H.Y., Kim Y.H., Nam J.Y., Kim B., Song G.S., Lim H.S., Bae K.S. (2021). Effect of Food on the Pharmacokinetics and Pharmacodynamics of a Single Oral Dose of Tegoprazan. Clin. Ther..

[5] Yang E., Kim S., Kim B., Kim B., Kim Y., Park S.S., Song G.S., Yu K.S., Jang I.J., Lee S. (2022). Night-time gastric acid suppression by tegoprazan compared to vonoprazan or esomeprazole. Br. J. Clin. Pharmacol..

[6] Han S., Choi H.Y., Kim Y.H., Choi S., Kim S., Nam J.Y., Kim B., Song G.S., Lim H.S., Bae K.S. (2023). Comparison of Pharmacodynamics between Tegoprazan and Dexlansoprazole Regarding Nocturnal Acid Breakthrough: A Randomized Crossover Study. Gut Liver.

[7] Jeong H.C., Kim M.G., Wei Z., Lee K.R., Lee J., Song I.S., Shin K.H. (2022). Integration of a Physiologically Based Pharmacokinetic and Pharmacodynamic Model for Tegoprazan and Its Metabolite: Application for Predicting Food Effect and Intragastric pH Alterations. Pharmaceutics.

[8] Ngo L.T., Lee J., Yun H.Y., Chae J.W. (2023). Development of a Physiologically Based Pharmacokinetic Model for Tegoprazan: Application for the Prediction of Drug-Drug Interactions with CYP3A4 Perpetrators. Pharmaceutics.

[9] Won Y.H., Yang J.H., Hong S.H., Goo Y.T., Lee S., Choi Y.W. (2023). Polymeric solid dispersion enhances the equilibrium solubility and oral bioavailability of tegoprazan. Bull. Korean Chem. Soc..

[10] Kang S., Won Y.-H., Yang J.-H., Byeon J., Lee S. (2026). Preparation and evaluation of tegoprazan tablets using long-term crystallization-suppressing solid dispersion technology. J. Pharm. Investig..

[11] Kim P., Lee I.-S., Kim J.-Y., Lee M.-J., Choi G.J. (2023). Amorphous solid dispersions of tegoprazan and three different polymers: In vitro/in vivo evaluation of physicochemical properties. Korean J. Chem. Eng..

[12] Bhujbal S.V., Mitra B., Jain U., Gong Y., Agrawal A., Karki S., Taylor L.S., Kumar S., Zhou Q.T. (2021). Pharmaceutical amorphous solid dispersion: A review of manufacturing strategies. Acta Pharm. Sin. B.

[13] Passerini N., Albertini B., Gonzalez-Rodriguez M.L., Cavallari C., Rodriguez L. (2002). Preparation and characterisation of ibuprofen–poloxamer 188 granules obtained by melt granulation. Eur. J. Pharm. Sci..

[14] Kukec S., Dreu R., Vrbanec T., Srcic S., Vrecer F. (2012). Characterization of agglomerated carvedilol by hot-melt processes in a fluid bed and high shear granulator. Int. J. Pharm..

[15] Shimpi S.L., Mahadik K.R., Paradkar A.R. (2009). Study on Mechanism for Amorphous Drug Stabilization Using Gelucire 50/13. Chem. Pharm. Bull..

[16] da Fonseca Antunes A.B., De Geest B.G., Vervaet C., Remon J.P. (2013). Gelucire 44/14 based immediate release formulations for poorly water-soluble drugs. Drug Dev. Ind. Pharm..

[17] Chaudhary R.S., Amankwaa E., Kumar S., Hu T., Chan M., Sanghvi P. (2016). Application of a hot-melt granulation process to enhance fenofibrate solid dose manufacturing. Drug Dev. Ind. Pharm..

[18] Yang D., Kulkarni R., Behme R.J., Kotiyan P.N. (2007). Effect of the melt granulation technique on the dissolution characteristics of griseofulvin. Int. J. Pharm..

[19] Sarabu S., Kallakunta V.R., Butreddy A., Janga K.Y., Ajjarapu S., Bandari S., Zhang F., Murthy S.N., Repka M.A. (2021). A One-Step Twin-Screw Melt Granulation with Gelucire 48/16 and Surface Adsorbent to Improve the Solubility of Poorly Soluble Drugs: Effect of Formulation Variables on Dissolution and Stability. AAPS PharmSciTech.

[20] Nyavanandi D., Narala S., Mandati P., Alzahrani A., Kolimi P., Almotairy A., Repka M.A. (2023). Twin Screw Melt Granulation: Alternative Approach for Improving Solubility and Permeability of a Non-steroidal Anti-inflammatory Drug Ibuprofen. AAPS PharmSciTech.

[21] Sadoqi M., Lau-Cam C.A., Wu S.H. (2009). Investigation of the micellar properties of the tocopheryl polyethylene glycol succinate surfactants TPGS 400 and TPGS 1000 by steady state fluorometry. J. Colloid Interface Sci..

[22] Sheu M.T., Chen S.Y., Chen L.C., Ho H.O. (2003). Influence of micelle solubilization by tocopheryl polyethylene glycol succinate (TPGS) on solubility enhancement and percutaneous penetration of estradiol. J. Control Release.

[23] Zhang Z., Tan S., Feng S.S. (2012). Vitamin E TPGS as a molecular biomaterial for drug delivery. Biomaterials.

[24] Lamm M.S., DiNunzio J., Khawaja N.N., Crocker L.S., Pecora A. (2016). Assessing Mixing Quality of a Copovidone-TPGS Hot Melt Extrusion Process with Atomic Force Microscopy and Differential Scanning Calorimetry. AAPS PharmSciTech.

[25] Krasavage W.J., Terhaar C.J. (1977). d-α-Tocopheryl Poly(ethylene glycol) 1000 Succinate. Acute Toxicity, Subchronic Feeding, Reproduction, and Teratologic Studies in the Rat. J. Agric. Food Chem..

[26] Dintaman J.M., Silverman J.A. (1999). Inhibition of P-Glycoprotein by D-α-Tocopheryl Polyethylene Glycol 1000 Succinate (TPGS). Pharm. Res..

[27] Collnot E.-M., Baldes C., Wempe M.F., Kappl R., Hüttermann J., Hyatt J.A., Edgar K.J., Schaefer U.F., Lehr C.-M. (2007). Mechanism of Inhibition of P-Glycoprotein Mediated Efflux by Vitamin E TPGS: Influence on ATPase Activity and Membrane Fluidity. Mol. Pharm..

[28] Jain S., Pandey S., Sola P., Pathan H., Patil R., Ray D., Aswal V.K., Bahadur P., Tiwari S. (2019). Solubilization of Carbamazepine in TPGS Micelles: Effect of Temperature and Electrolyte Addition. AAPS PharmSciTech.

[29] Sotthivirat S., Ramesh R., Wasylaschuk W., Bottone C., Xia B., Stellabott J., McNevin M., Skomski D., Brown C. (2020). Effect of TPGS surfactant on dissolution sensitivity of a poorly water-soluble drug using high-shear wet granulation. Powder Technol..

[30] Raut S., Karzuon B., Atef E. (2015). Using in situ Raman spectroscopy to study the drug precipitation inhibition and supersaturation mechanism of Vitamin E TPGS from self-emulsifying drug delivery systems (SEDDS). J. Pharm. BioMed Anal..

[31] Oberle R.L., Moore T.J., Krummel D.A.P. (1995). Evaluation of Mucosal Damage of Surfactants in Rat Jejunum and Colon. J. Pharmacol. Toxicol. Methods.

[32] Lerk C.F., Bolhuis G.K., de Boer A.H. (1979). Effect of Microcrystalline Cellulose on Liquid Penetration in and Disintegration of Directly Compressed Tablets. J. Pharm. Sci..

[33] Yassin S., Goodwin D.J., Anderson A., Sibik J., Wilson D.I., Gladden L.F., Zeitler J.A. (2015). The Disintegration Process in Microcrystalline Cellulose Based Tablets, Part 1: Influence of Temperature, Porosity and Superdisintegrants. J. Pharm. Sci..

[34] Conti S., Maggi L., Segale L., Ochoa Machiste E., Conte U., Grenier P., Vergnault G. (2007). Matrices containing NaCMC and HPMC 1. Dissolution performance characterization. Int. J. Pharm..

[35] Van Eerdenbrugh B., Vercruysse S., Martens J.A., Vermant J., Froyen L., Van Humbeeck J., Van den Mooter G., Augustijns P. (2008). Microcrystalline cellulose, a useful alternative for sucrose as a matrix former during freeze-drying of drug nanosuspensions—A case study with itraconazole. Eur. J. Pharm. Biopharm..

[36] Guo K., He X., Zhao H., Ma C. (2023). Characterisation of degradation products of tegoprazan by LC-MS and GC-MS. J. Pharm. BioMed Anal..

[37] Tatavarti A., Kesisoglou F. (2015). An Extrusion Spheronization Approach to Enable a High Drug Load Formulation of a Poorly Soluble Drug with a Low Melting Surfactant. J. Pharm. Sci..

[38] Liu Z., Lansley A.B., Duong T.N., Smart J.D., Pannala A.S. (2022). Increasing Cellular Uptake and Permeation of Curcumin Using a Novel Polymer-Surfactant Formulation. Biomolecules.

[39] Song I.S., Cha J.S., Choi M.K. (2016). Characterization, in Vivo and in Vitro Evaluation of Solid Dispersion of Curcumin Containing d-alpha-Tocopheryl Polyethylene Glycol 1000 Succinate and Mannitol. Molecules.

[40] Tarchoun A.F., Trache D., Klapotke T.M. (2019). Microcrystalline cellulose from Posidonia oceanica brown algae: Extraction and characterization. Int. J. Biol. Macromol..

[41] Tran P.H.-L., Tran T.T.-D., Park J.B., Min D.H., Choi H.G., Han H.K., Rhee Y.S., Lee B.J. (2011). Investigation of physicochemical factors affecting the stability of a pH-modulated solid dispersion and a tablet during storage. Int. J. Pharm..

[42] Moore J.W., Flanner H.H. (1996). Mathematical comparison of curves with an emphasis on in vitro dissolution profiles. Pharm. Technol..

[43] Shah V.P., Tsong Y., Sathe P., Liu J.-P. (1998). In Vitro Dissolution Profile Comparison—Statistics and Analysis of the Similarity Factor, f2. Pharm. Res..

[44] Xu M., Cai Y., Wang Y., Lin M., Weng Q. (2025). A Novel UPLC-MS/MS Method for Determining Tegoprazan in Rat Plasma: An Application in a Rat Pharmacokinetic Study. BioMed Chromatogr..

